# Hospitalization Trends and Healthcare Resource Utilization for Fecal Impactions in Pediatric Patients with Functional Constipation

**DOI:** 10.3390/jcm14020569

**Published:** 2025-01-17

**Authors:** Diem Le, Hafiza Durrani, Jasmine Khatana, Sujithra Velayuthan, Senthilkumar Sankararaman, Aravind Thavamani

**Affiliations:** 1 Department of Pediatrics, MetroHealth Medical Center, Cleveland, OH 44109, USA; dle@metrohealth.org (D.L.);; 2 Division of Pediatric Neurogastroenterology and Motility, Nationwide Children’s Hospital, Columbus, OH 43205, USA; 3 Division of Pediatric Gastroenterology, Hepatology and Nutrition, UH Rainbow Babies and Children’s Hospital, Case Western Reserve University, Cleveland, OH 44106, USA; drsskumar@gmail.com

**Keywords:** fecal impaction, functional constipation, hospitalization, healthcare resource utilization, population-based analysis, pediatric patients

## Abstract

**Objectives**: To analyze the clinical characteristics, trends in hospitalization, and healthcare resource utilization of pediatric patients with fecal impaction. **Methods**: We utilized the Healthcare Cost and Utilization Project (HCUP) databases, including the National Inpatient Sample (NIS) and the Kids Inpatient Database (KID) datasets from 2011 to 2019, to include all hospitalizations of patients up to 18 years of age with a primary diagnosis of (1) fecal impaction or (2) a primary diagnosis of abdominal pain or constipation with a secondary diagnosis of fecal impaction. The study analyzed various comorbid factors and clinical characteristics of these patients. For healthcare resource utilization, we analyzed the length of hospital stays and total hospital charges, adjusted for inflation. **Results**: A total of 23,570 admissions due to fecal impactions in children between the years 2011 and 2019 were analyzed, contributing to 0.18% of the total pediatric admissions. Hospitalization rates nearly doubled from 2011 (0.15%) to 2019 (0.29%). The mean hospitalization charges also trended upwards from 15,234 USD in 2011 to 22,487 USD in 2019. The inflation-adjusted annual rate of increase in hospital charges during this period was 5.9% per year. Aggressive fecal disimpaction procedures (either manual or surgical) were performed in approximately 3% of these admissions. Multivariate regression showed that older children (13–18 years of age) were more likely to require aggressive disimpaction. Female children, those with Hispanic ethnicity, and those with obesity were less likely to be associated with the need for disimpaction. **Conclusions**: Hospitalizations for fecal impaction have increased significantly over the past decade, creating a substantial burden on healthcare resources. Our study highlights the importance of aggressive outpatient management strategies with close follow-up for fecal impactions, which will potentially minimize these hospitalizations.

## 1. Introduction

Childhood functional constipation is characterized by reduced frequency of stooling and/or the passage of painful, hard stools and can be associated with retentive fecal incontinence [1]. It is a common problem, with an estimated median global prevalence of 8.9–14% [2,3,4] In the United States, the prevalence of constipation in children was estimated to vary between 0.7% and 29.6% [5].

Children with constipation have significantly higher healthcare utilization compared to their peers without constipation, as evidenced by increased clinic visits, emergency department visits, and inpatient hospitalizations [6]. Beyond the heavy financial burden on the healthcare system, constipation also adversely impacts the health-related quality of life in children [7]. The mainstay of constipation management includes educating parents and caregivers, administering osmotic laxatives, such as polyethylene glycol (PEG 3350) or lactulose, on a daily basis, and implementing behavioral modification, like scheduled sitting on the commode after meals [8].

Fecal impaction, a common yet potentially serious disorder, occurs when hardened stool is retained in the large intestine and cannot be expelled through regular bowel movements [9]. This large fecal mass is noted either in abdominal palpation or per rectal examination [10]. In rare cases, an abdominal radiograph may be required to evaluate for disimpaction in children when a physical examination is not possible or reliable. Even though fecal impaction can occur in all ages, children are particularly vulnerable [9]. Fecal impaction is unlikely to be resolved with just maintenance laxative regimens, such as low-dose oral polyethylene glycol (PEG) 3350 and lactulose. Severe fecal impaction often warrants inpatient admission for fecal disimpaction management. Fecal disimpaction modalities include the administration of frequent enemas or suppositories, higher doses of PEG 3350, or management with manual removal of the fecal mass [10,11]. Unfortunately, a significant proportion of children receive suboptimal outpatient therapy prior to hospital admissions, even though a previous quality improvement study noted that aggressive outpatient management has reduced inpatient hospitalizations [8,12].

In a single-center, retrospective pediatric study involving 188 patients, approximately 70% of the cohort was successfully managed on an outpatient basis, while the remaining patients were admitted during the two-year study period [13]. The authors noted that the inpatient group tended to be younger (median age of 3.6 years in the cohort who were admitted versus five years in the outpatient cohort), were predominantly of Black race, and had various comorbidities, such as prematurity and developmental delay [13].

Healthcare costs for constipated children are 2–4 times higher than those for the general pediatric population, with an additional 3.9 billion USD/year for children with constipation based on the Medical Expenditure Panel Survey (MEPS) in 2003–2004 [6,14]. Children with constipation require frequent clinic visits, emergency department visits, and inpatient admissions for their recurrent symptoms [15,16]. Particularly, inpatient care accounts for 55% of the total cost of treating constipation, despite most cases being managed in ambulatory settings [17]. In a retrospective study by Stephens et al. involving a large sample of 4.9 million children from ten states of the United States between 2009 and 2011, 5.4% of children had the diagnosis of constipation and the total expenditure was approximately 80 million USD [12]. Outpatient management accounted for 84% of this spending (about 67 million USD), while only 0.5% (N = 1363) required inpatient management but accounted for 15.4% (approximately 12 million USD) of the total cost. The mean spending was 7815 USD per hospital stay and 40.5% of these hospitalized children did not have an outpatient visit for constipation before admission. Similarly, another study by Martin et al. found that only 1.6% of patients with constipation were treated in inpatient settings, but they accounted for nearly 55% of the total costs of constipation management [18].

Currently, functional constipation is considered a global public health problem exacerbated by rising risk factors for constipation such as obesity, increased consumption of processed foods, reduced fiber intake, and changes in family social structures [1,19,20,21,22,23]. These factors are expected to contribute to an increased prevalence of constipation in the future, leading to a proportional increase in hospital admissions for fecal impaction and a consequent rise in healthcare costs [7,19,24,25]. Various studies have highlighted the growing healthcare expenditure associated with increased outpatient and emergency department visits for pediatric patients with constipation [3,15].

Despite being a global issue, there is a paucity of national-level epidemiological data on admission rates and healthcare resource utilization for children admitted for the management of fecal impaction and their trends over the years. In this study, we hypothesized that the incidence rate of inpatient management of fecal impaction could be increasing. This study aimed to evaluate hospitalization rates and healthcare resource utilization for children hospitalized due to fecal impaction from 2011 to 2019 at a national level. Our findings aim to equip physicians and policymakers with data to estimate this burden, aiding in the development of preventive strategies and healthcare policies to improve patient care and optimize healthcare resources.

## 2. Materials and Methods

We utilized the Healthcare Cost and Utilization Project (HCUP) databases, specifically the National Inpatient Sample (NIS) and the Kids Inpatient Database (KID), for the years 2011 to 2019.

The KID database contains data on hospitalized patients under 21 years old, based on discharge abstracts for billing, and is the largest publicly available all-payer pediatric inpatient care database in the United States. Data are released approximately every three years, and the data for 2012, 2016, and 2019 were included in our study (data for 2015 was not available due to the transition from the International Classification of Diseases (ICD)-9 to ICD-10).

The NIS database is the largest publicly available all-payer inpatient healthcare database, designed to generate U.S. regional and national estimates of inpatient utilization, access, cost, quality, and outcomes. The NIS collects inpatient data, which are sampled at a rate of 20% from data covering more than 97% of the U.S. population. Released annually, it contains records for about seven million hospitalizations annually and is designed to provide national-level estimation. We used the NIS database for the remaining years (2011, 2013–2015, 2017, and 2018) when KID data was not available. As KID data exclusively include pediatric patients, are sampled at a rate of 80%, and are released approximately once every three years, we employed KID data for the years available, as noted previously.

### 2.1. Study Population

We included pediatric patients (aged under 18 years) from the datasets with a primary diagnosis of fecal impaction using the ICD-9 code (560.32), ICD-10 code (K56.41), a primary diagnosis of abdominal pain (R10.9, R10.30, R10.31, R10.32, R10.33, and R10.84), or constipation (K59.0) with a secondary diagnosis of fecal impaction at discharge. Patients under 1 year of age were excluded as this group is less likely to experience hospitalization for fecal disimpaction and has more potential to have surgical causes of constipation. Furthermore, hospital admissions during infancy may further underestimate the hospitalization rates for fecal disimpaction in this age group.

Cases of non-functional constipation due to underlying conditions were excluded to minimize confounding. These conditions included celiac disease, hypothyroidism, hypercalcemia, hypokalemia, dietary protein allergy, vitamin D intoxication, cystic fibrosis, Hirschsprung disease, anal achalasia, colonic inertia, anal stenosis, imperforate anus, pelvic mass, spinal cord anomalies, abnormal abdominal musculature, pseudo-obstruction, multiple endocrine neoplasia, and drug or toxic exposure (e.g., opiates, antidepressants, chemotherapy, or lead ingestion).

### 2.2. Study Variables

We collected demographic characteristics such as age, sex, race/ethnicity, type of insurance, and various comorbid factors. The hospitalization data, including annual hospitalization rates, length of stay (LOS) in days, and total hospital charges in USD, were obtained from the HCUP-KID dataset. Information on fecal disimpaction methods, including both manual and surgical techniques, was also extracted for analysis.

Age groups were categorized into three ranges: <5 years, 5–12 years, and 13–18 years. Racial/ethnic categories included White, Black, Hispanic, and Others. Insurance types were classified as public (Medicare or Medicaid), private (commercial insurance or health maintenance organizations), and uninsured or self-pay/others. We analyzed and compared various demographic and clinical characteristics among patients with and without the need for inpatient fecal disimpaction procedures (manual or surgical).

### 2.3. Statistical Analysis

Demographic characteristics were summarized as mean ± standard deviation (SD) for continuous variables and as percentages for categorical variables. The chi-squared test was employed to assess differences in categorical data across various groups, and the Student’s *t*-test was used to compare continuous variables. Primary outcome measures, reported as percentages over the study period, included hospitalization rates and trends. LOSs and total charges were analyzed and reported as means ± SD for healthcare resource utilization.

Trend analysis was completed using weighted least squares regression analysis. The total charges were adjusted for inflation to the year 2019 using the consumer price index (calculator) from the United States Department of Labor. A multivariate logistic regression model was developed to evaluate various factors associated with fecal disimpaction procedures, including all factors with a *p* value < 0.2 in univariate analysis. A *p* value < 0.05 was considered statistically significant. All statistical analyses were conducted using SPSS version 24.

## 3. Results

A total of 23,570 pediatric hospitalizations due to fecal impaction in children from 2011 to 2019 were analyzed, contributing to 0.18% of all pediatric admissions. The demographic details are summarized in Table 1. Approximately half of the cohort was between 5 and 12 years of age, followed by children less than 5 years (28.5%) and those between 13 and 18 (28.3%) years. Males comprised 55% of the cohort, with females accounting for 45%. About half of the patients were White, followed by Hispanic and Black race/ethnicity. Around 62% of patients had public payor insurance, 31.45% had private insurance, and 6.7% were self-pay/others.

**Figure 1:**
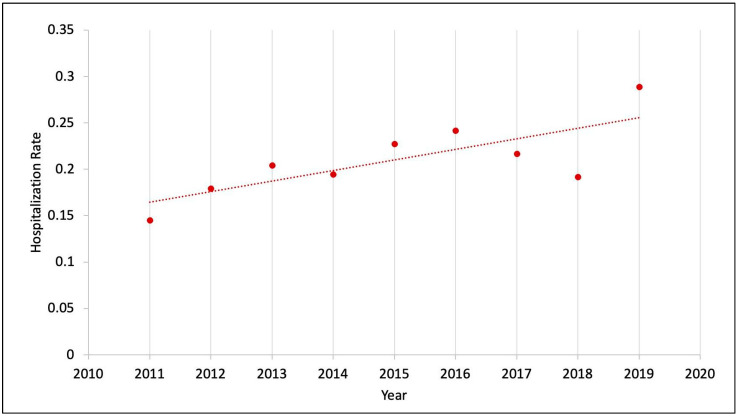
Trend of hospitalization rates from 2011 to 2019.

Trend in hospitalization due to fecal impaction: The hospitalization rate due to fecal impaction was 1.5 per 1000 total pediatric admissions in 2011 and nearly doubled to 2.9 per 1000 total pediatric admissions in 2019. There was a significant uptrend from 2011 to 2019 based on trend analysis with *p* value < 0.01 (Figure 1).

**Figure 2:**
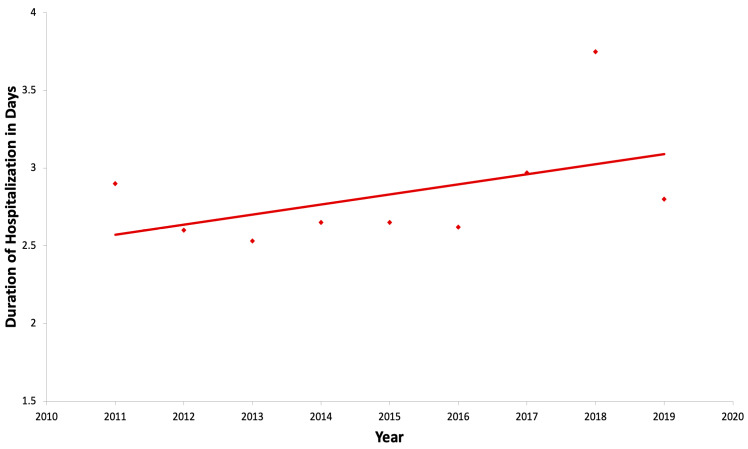
Trend of mean length of hospital stays from 2011 to 2019.

Trend in healthcare resource utilization: The length of hospital stays for children with functional constipation hospitalized due to fecal impaction varied from 2.5 to 3.7 days. The shortest mean length of stay was 2.5 days, observed in 2013, and the longest mean length of stay was 3.7 days, noted in 2018. However, overall, there was no statistically significant change in trend analysis with *p* = 0.45 (Figure 2).

**Figure 3:**
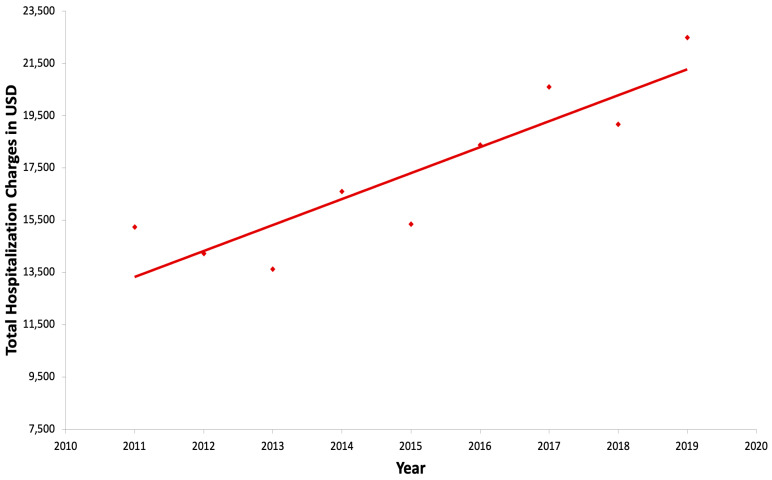
Trend of total hospitalization charges from 2011 to 2019.

The mean hospitalization charge trended up from 15,234 USD in 2011 to 22,487 USD in 2019 and was statistically significant with a *p* value < 0.01 in trend analysis. The inflation-adjusted annual rate of increase in hospital charges during this period was 5.9% per year (Figure 3).

Variables associated with fecal disimpaction procedures: Nearly 3% (N = 690) of these admissions due to fecal impaction in children with functional constipation required manual or surgical disimpaction.

In univariate analysis, the group requiring disimpaction procedures was older (from 13 to 18 years of age), predominantly male, of White race, and had slightly higher public insurance rates (Table 1). Those of the female sex, of Hispanic ethnicity, and with obesity (data not included in Table 2) were less likely to be associated with aggressive disimpaction (*p* < 0.001). No significant association was found between insurance type and procedure group (*p* = 0.16). 

In multivariate regression (data not included), we found that older children (from 13 to 18 years of age) were more likely to require more aggressive disimpaction procedures (*p* < 0.001). However, gender, race/ethnicity, and insurance status were not significantly associated with the need for aggressive disimpaction procedures.

## 4. Discussion

Our study is one of the largest population-based studies analyzing clinical characteristics, hospitalization trends, healthcare costs, and comorbidities associated with fecal impaction in children with functional constipation. Our study highlighted the rising burden of fecal impaction on inpatient healthcare, contributing to a substantial rise in healthcare expenditure and negative impacts on families.

We included a sample of 23,570 pediatric hospitalizations due to fecal impaction in children from 2011 to 2019, contributing to 0.18% of the total pediatric admissions. Using a different national-level database, the Pediatric Health Information System (PHIS), Librizzi et al. reported 12,804 patients hospitalized with functional constipation and a prevalence of 0.65%, ranging from 0.19 to 1.41% among individual hospitals [11]. In a study from Australia, hospital prevalence of pediatric constipation among total hospitalizations was approximately 1% [15]. These epidemiological differences can be explained by the methodological differences used between the studies.

The demographic distribution we observed was similar to that in the national-level database study by Librizzi et al. [11]. Liem et al. found no significant differences regarding age, sex, race, and socioeconomic status between children with and without functional constipation [17]. However, a more recent meta-analysis showed that female gender, increasing age, and socioeconomic status adversely affected constipation prevalence rates [5]

Regarding age, our study revealed that children younger than 13 years constituted approximately three-fourths of the cohort, but older children aged 13 to 18 years required manual or surgical disimpaction procedures at higher rates than those receiving medications only. According to Sinha et al. [13], there was no association between age at diagnosis of constipation in pediatric patients with fecal impaction and the need for inpatient treatment, but an earlier age of onset was more likely to require inpatient treatment. Due to limited data, further follow-up studies are needed to evaluate the relationship between age at diagnosis of fecal impaction and response to treatment in children.

The evidence of sex/gender differences in the demographic characteristics of constipation is inconsistent in children, unlike the female preponderance in adults [4,6,25,26,27,28,29,30]. We noted that the female sex was less likely to be associated with the need for aggressive disimpaction procedures (*p* < 0.001), while there were no differences associated with sex between the inpatient treatment and outpatient treatment groups in the above study [13]. A study in 2022 using computed tomographic colonography revealed that female colons are significantly longer than male colons, and the proportion of patients with daily bowel habits was significantly lower in females compared with males [31]. However, this does not explain why the female gender was less likely to be associated with aggressive disimpaction needs than males in our study. It is possible that female patients were more inclined to seek medical care sooner, preventing progression to severe impaction, but this hypothesis requires further investigation.

We noted that Hispanic ethnicity was associated with a decreased need for disimpaction procedures in univariate analysis. A retrospective study comparing characteristics of patients with fecal impaction who received outpatient disimpaction successfully versus those who had failed outpatient treatment and needed further inpatient management found that Hispanic children had a larger proportion in outpatient treatment compared to inpatient treatment (*p* = 0.019), whereas Black patients had a larger proportion in the inpatient treatment group as compared to the outpatient group (*p* < 0.001) [13]. In another study, the authors noted that Black families were less likely to report constipation symptoms to their primary pediatrician, which might contribute to more severe constipation and a lower likelihood of receiving optimal outpatient management [32]. This association between race/ethnicity and the need for inpatient management as well as aggressive disimpaction procedures needs further evaluation.

The pattern of insurance types we noted was similar to the NIS database study described by Sethi et al. [33]. In our study, no association was found between insurance type and the need for aggressive disimpaction procedures. We could not identify other studies that either confirm or refute these findings. However, Sinha et al. also found no association between insurance types and inpatient versus outpatient treatment groups in their study [13].

Some studies found that constipation is more prevalent in obese patients, but there is a lack of data about the association between obesity and treatment failure for constipation or fecal impaction [34,35,36]. Our data supported that patients with obesity were less likely to require disimpaction procedures. Further, prospective studies should evaluate this association to elucidate the underlying reasons for this pattern.

Trends in hospitalization rates: Our study demonstrated a notable upward trend in the rate of hospitalization due to fecal impaction among pediatric admissions. Between 2011 and 2019, the incident rate nearly doubled, increasing from 1.5 per 1000 admissions to 2.9 per 1000 admissions (Figure 1). This trend largely followed a stable trend line, except for an outlier year in 2018. This rising trend may reflect an actual increase in constipation prevalence, predisposing children to more frequent fecal impactions, and/or indicate an increase in suboptimal outpatient management, resulting in higher rates of hospitalizations. Supporting these findings, Sethi et al. [33] analyzed the National Inpatient Sample (NIS) database and observed a similar trend between 1997 and 2010, during which hospitalizations for constipation among children aged 1–17 years increased from 1.65 per 1000 admissions in 1997 to 5 per 1000 admissions in 2010.

Trend in healthcare resource utilization: The mean length of hospital stays for children with functional constipation hospitalized due to fecal impaction in our study ranged from 2.5 to 3.7 days. The shortest mean length of stay, at 2.5 days, was noted in the year 2013, and the longest mean length of stay, at 3.7, was noted in the year 2018. However, no statistically significant trend was observed in the length of stay over the study period with *p* = 0.45 (Figure 2). The duration of stay in our study was somewhat comparable to other studies [15,33]. For instance, Ansari et al. reported a mean hospital stay of 4.4 days among 8688 pediatric admissions for constipation [15]. In the study by Sethi et al., the mean hospitalization duration slightly increased, from 3 to 3.1 days, during the study period from 1997 to 2010 [33]. The variations in the length of stay noted in these studies may be reflected by differences in methodology, such as criteria for hospitalization and inclusion or exclusion parameters.

In our study, the mean hospitalization charges for pediatric admissions due to fecal impaction trended up from 15,234 USD in 2011 to 22,487 USD in 2019 and was statistically significant with a *p* < 0.01 in trend analysis. This trend remained significant even after adjusting for inflation. Also, the trend in the charges was statistically significant despite the absence of a statistically significant trend in the length of hospital stays, suggesting that other factors may have contributed to the increased costs. Potential contributors to this trend include variations in clinical coding practices, changes in the reimbursement policies by payers, and differences in treatment modalities, such as procedural disimpaction versus medication disimpaction. In a pediatric study by Stephens et al. [12], the average inpatient expenditure for constipation was 7815 USD per hospitalization. In the study utilizing the NIS, in both children and adults, the authors noted that the mean hospital charges were 8869 USD (adjusted for inflation) in 1997 which increased significantly to 17,518 USD in the year 2010, *p* < 0.001 [33]. The differences in hospital expenditure between these studies are likely due to different methodologies used. Our study revealed an inflation-adjusted annualized rate of increase in hospital charges during this period was 5.9% per year (Figure 3). This increase in inflation-adjusted hospitalization charges, despite a largely stable length of stay, also highlights the rising costs of healthcare in the United States.

This study has various limitations. As a retrospective study, it is thus subject to the inherent limitations of such studies. Coding errors are inevitable in large datasets such as those used in this study and should be considered while reviewing the results due to the reliance on coding accuracy to diagnose functional constipation and related conditions. The admission rates and healthcare expenditures for fecal impaction may be underestimated due to the study’s attempts to accurately capture admissions with fecal impactions only and not including constipation alone as an inclusion criterion. The lack of outpatient data limited us from exploring the use of outpatient services, prior gastroenterology consultations, home disimpaction management, and other preventive strategies to limit the progression to severe fecal impaction requiring admission. Similarly, we did not have information on whether patients were directly admitted or admitted after emergency department evaluation. Finally, comorbid factors, such as autism and attention deficit hyperactive disorder which could predispose inpatient hospitalization for fecal impaction, were not evaluated.

Despite these limitations, this study possesses numerous strengths. We provided national-level longitudinal data with detailed demographics of the children admitted with fecal impactions between 2011 and 2019 while also demonstrating an increasing trend in hospitalization rates and charges during the study period. The hospitalization charges associated with inpatient admission presented a significant upward trend after adjusting for inflation, highlighting the opportunity for improved screening and aggressive outpatient management of constipation to minimize healthcare expenditure. These findings shall emphasize to clinicians, policymakers, and other stakeholders that focusing on aggressive outpatient preventive strategies, including family education (advice on a healthy diet, fluid intake, and toilet training), management of constipation, closer outpatient follow-ups, and early referral to pediatric gastroenterologists, may reduce the incidence rate of fecal disimpaction requiring hospitalization.

## 5. Conclusions

Hospitalization due to fecal impaction in children with functional constipation has been overall increasing during the past decade, posing a considerable strain on healthcare utilization with upwards-trending inflation-adjusted total hospital charges. This study highlights the need for aggressive outpatient management by devising protocols, screening at-risk patients, and frequent follow-up, which could decrease the admissions for fecal impactions and, subsequently, healthcare. We encourage more studies to further explore this trend and related factors to identify effective preventive strategies.

## Figures and Tables

**Table 1:** Demographic details of the entire cohort during the study period.

Parameters	Total 23,570
Age	
<5 Years	6722 (28.5%)
5 to 12 years	10,154 (43%)
13 to 18 years	6691 (28.3%)
Gender	
Female	10,601 (44.9%)
Male	12,924 (54.8%)
Race/Ethnicity	
White	11,508 (51.5%)
Black	3812 (17%)
Hispanic	5758 (25.8%)
Others	1232 (5.5%)
Insurance	
Public	14,544 (61.7%)
Private	7417 (31.4%)
Self-pay/others	1587 (6.7%)

**Table 2:** Demographic differences between children admitted for fecal impaction who required disimpaction procedures versus medications (univariate analysis).

Parameters	Manual or Surgical Disimpaction *n* = 690	Medication Only *n* = 22,880	*p* Value
Age			<0.001
<5 Years	152 (22.1%)	6570 (28.7%)	
5 to 12 years	265 (38.5%)	9889 (43.2%)	
13 to 18 years	272 (39.4%)	6419 (28.1%)	
Gender			<0.001
Female	256 (37.1%)	10,345 (45.3%)	
Male	434 (62.9%)	12,490 (54.7%)	
Race/Ethnicity			0.001
White	371 (57%)	11,137 (51.4%)	
Black	115 (17.7%)	3697 (17.1%)	
Hispanic	125 (19.2%)	5633 (26%)	
Others	40 (6.1%)	1192 (5.5%)	
Insurance			0.16
Public	437 (63.3%)	14,107 (61.7%)	
Private	198 (28.7%)	7219 (31.6%)	
Self-pay/others	55 (8%)	1532 (6.7%)	

## Data Availability

Publicly available datasets were analyzed in this study. This data can be found here: https://hcup-us.ahrq.gov/databases.jsp (accessed on 28 November 2024).
